# Subacute exposure to apigenin induces changes in protein synthesis in the liver of Swiss mice

**DOI:** 10.3389/fphys.2025.1576310

**Published:** 2025-05-09

**Authors:** Łukasz S. Jarosz, Katarzyna Socała, Katarzyna Michalak, Kamila Bulak, Artur Ciszewski, Agnieszka Marek, Zbigniew Grądzki, Piotr Wlaź, Edyta Kowalczuk-Vasilev, Anna Rysiak

**Affiliations:** ^1^ Department of Epizootiology and Clinic of Infectious Diseases, Faculty of Veterinary Medicine, University of Life Sciences in Lublin, Lublin, Poland; ^2^ Department of Animal Physiology and Pharmacology, Institute of Biological Sciences, Faculty of Biology and Biotechnology, Maria Curie–Skłodowska University, Lublin, Poland; ^3^ Department of Pathomorphology and Forensic Veterinary Medicine, Faculty of Veterinary Medicine, University of Life Sciences in Lublin, Lublin, Poland; ^4^ Department of Preventive Veterinary and Avian Diseases, Faculty of Veterinary Medicine, University of Life Sciences in Lublin, Lublin, Poland; ^5^ Institute of Animal Nutrition and Bromatology, Faculty of Animal Science and Bioeconomy, University of Life Sciences in Lublin, Lublin, Poland; ^6^ Department of Botany, Mycology, and Ecology, Maria Curie-Skłodowska University, Lublin, Poland

**Keywords:** apigenin, mouse, liver histology, proteomic profile, MALDI-TOF MS, cytokine concentration

## Abstract

Apigenin is a natural flavonoid with various pharmacological properties. Available data indicate that it affects the metabolic processes and protein profile of cells, including hepatocytes. However, there is speculation that the use of apigenin may have a hepatotoxic effect. The aim of the experiment was to assess the effect of apigenin administered intraperitoneally to mice on the concentrations of pro- and anti-inflammatory cytokines in the liver tissue and to analyse liver weight and morphological changes in the liver parenchyma. A proteomic analysis was also performed to examine differences in genes expression for specific proteins in liver cells. Adult male albino Swiss mice were divided into two groups and treated with either apigenin (50 mg/kg BW) – APG, or a vehicle (1% DMSO) – CONT, every 24 h for 14 days. The material for the study consisted of liver samples. Slight hepatocyte degeneration microscopically were demonstrated in most mice exposed to apigenin. No significant differences were observed in the absolute and relative weight of the liver or the concentrations of pro- and anti-inflammatory cytokines between the control and experimental group. The mass spectrometry results indicate significantly higher synthesis of the proteins MAP2K19, CEP69, GNMT, BPIFA3, SYT17, ANKRD1, GRHPR, CLEC1A and EF2 in the livers of mice from the APG group in comparison to CONT group. Exposure of mice to apigenin induces functional changes in the liver. In conjunction with the microscopical and proteomic analyses, this study may indicate that inflammatory changes developing in the liver could be self-limiting and subject to regenerative processes.

## 1 Introduction

The effect on liver cells exerted by various exogenous and endogenous factors in humans and animals, including those associated with the obesity, excessive alcohol consumption and the use of drugs also promotes the development of inflammation and disturbances of the oxidant/antioxidant balance, leading to hepatocyte damage and the development of acute liver failure (Bi et al., 2023; Devarbhavi et al., 2023; David and Hamilton, 2010). Apoptosis-resistant hepatic stellate cells (HSCs) are stimulated during these processes, leading to fibrosis, cirrhosis, and even hepatocellular carcinoma (HCC) (Czech et al., 2013). The numerous limitations associated with traditional drug therapy for liver disease have necessitated the search for new compounds supporting treatment or providing alternative treatment methods. These compounds currently include apigenin (APG – 4,5,7-trihydroxyflavone) flavonoid present in fruit (oranges and nuts), vegetables (celery and parsley), and herbs (chamomile and coriander) (Tsanova-Savova and Ribarova, 2013; Mushtaq et al., 2023). The results of numerous studies indicate that apigenin has anti-tumour (Imran et al., 2020), anti-inflammatory, antiproliferative, antibacterial, and antioxidant properties (Mushtaq et al., 2023; Kowalski et al., 2005; Ginwala et al., 2019). In human medicine, apigenin is used in the prevention and treatment of cancers, especially of the respiratory system, due to its cytostatic and/or cytotoxic effect on cancer cells (Shukla and Gupta, 2010; Oishi et al., 2013). In addition, this compound has been shown to protect against the development of pancreatitis, to protect the nerve cells, and to prevent cardiovascular disease by directly lowering blood pressure (Zhu et al., 2016). Apigenin also inhibits the inflammatory response induced by LPS by inhibiting the synthesis of pro-inflammatory cytokines such as TNF- α, IL-6, and IL-1β (Patil et al., 2016; Zhou et al., 2017).

Animal studies have shown that apigenin exhibits protective activity in various models of liver dysfunction induced by substances such as acetaminophen, furan, and N-nitrosodiethylamine (NDEA) (Yang et al., 2013; Wang et al., 2014). Administration of apigenin to rats following the application of the carcinogen N-nitrosodiethylamine, which leads to liver damage and fibrosis (Bartsch et al., 1989), reduces concentrations of liver enzymes, increases free radical scavenging, and reduces lipid peroxidation in the liver, thereby preventing damage to the cell membrane of hepatocytes (Bartsch et al., 1989; Ali et al., 2014). The use of apigenin in rats fed a low-protein diet and additionally receiving acetaminophen (APAP) protects the liver against focal necrosis with diffuse infiltration of inflammatory cells (Mohamed et al., 2020). The use of apigenin, however, may also have negative consequences, as it generates reactive oxygen species (ROS) and phenoxyl radicals, which damage cells by increasing lipid peroxidation in the liver or inducing changes in gene expression (Kachadourian and Day, 2006; Andueza et al., 2015). Previously published findings concern the effect of apigenin administered a single time by various routes. A single dose of apigenin administered intraperitoneally at 25 or 50 mg/kg BW was shown to have no toxic effect in mice (Singh et al., 2012). At higher doses, however, e.g., 100 or 200 mg/kg BW, it leads to liver damage, expressed as elevated serum concentrations of alanine aminotransferase (ALT), aspartate aminotransferase (AST), and alkaline phosphatase (ALP), increased ROS concentrations, and stimulation of lipid peroxidation (Singh et al., 2012). High single doses of apigenin cause a decrease in SOD (superoxide dismutase) and GST (glutathione S-transferase) activity and glutathione (GSH) levels and an increase in CAT (catalase) and GPx (glutathione peroxidase) activity, as well as a decrease in the mRNA expression of Hsp70 (heat shock protein 70) in the liver (Singh et al., 2012). In addition, apigenin causes histopathological changes in the liver, such as hydropic changes or ballooning degeneration of the hepatocytes. Independently of these processes, high doses of apigenin affect various genes taking part in the regulation of cell growth and the intensity of stress reactions and apoptosis, increasing apoptosis of liver cells damaged by the products of fat or protein oxidation or DNA degradation (Vargo et al., 2006). These findings clearly indicate that high doses of apigenin induce oxidative stress, disturbing the oxidant/antioxidant balance, which is associated with hepatotoxic effects leading to liver failure.

It has been shown that the use of apigenin during the development of inflammation in the liver reduces the synthesis of proinflammatory cytokines TNF-α, IL-1, IL-6, IL-17 and IFN-γ, secreted as a result of the activation of numerous signaling pathways, especially NF-κB (nuclear factor kappa-light-chain-enhancer of activating B cells), a protein complex acting as a transcription factor (Ginwala et al., 2019). Various properties of apigenin and its effects on the cell lead to the activation of various processes and metabolic pathways. Literature data indicate that the use of apigenin increases or decreases the expression of genes in cells, resulting in increased synthesis or breakdown of proteins (Subhasitanont et al., 2017). For example, in apoptosis of cancer cells, apigenin causes an increase in the synthesis of proteins S100-A6 and S100-A11, thereby inhibiting the development of the neoplastic process and reducing the aggressiveness of non-apoptotic tumour cells (Subhasitanont et al., 2017).

Available literature data on the effects and mechanisms of action of apigenin clearly indicate that it affects the protein profile of cells, including hepatocytes, and has secondary effects on metabolic processes in states of health and disease (Hsu et al., 2021; Chen et al., 2023). At the same time, the literature includes no studies of the effect of the use of apigenin on the pattern of genes expression in the liver. With this in mind, we hypothesized that the use of apigenin in mice may increase the synthesis of proteins affecting metabolic processes in the liver, modulate the immune response, and cause changes in the histoarchitecture of the liver, which in the long term can lead to liver failure. To verify this hypothesis, we conducted a study to assess the effect of apigenin on the serum concentrations of liver enzymes, blood parameters, and the concentrations of pro- and anti-inflammatory cytokines TNF-α, IL-1β, IFN-γ, IL-6, IL-18, IL-10 and TLR4 (toll-like receptor 4) in the liver tissue of mice. In addition, microscopic evaluation of H&E-stained liver tissue samples was performed to show morphological changes in the liver tissue. The body weight of the animals, the weight of the liver, and the ratio of liver weight to body weight were determined as well. Proteomic analysis was also performed to investigate differences in the synthesis of proteins in the liver cells of mice treated with apigenin in comparison to the control group.

## 2 Materials and methods

### 2.1 Animals

Twenty adult male albino Swiss mice, obtained from an accredited breeder (Laboratory Animal Breeding, Ilkowice, Poland) at the age of 4–5 weeks, were used in the study. Upon arrival, the animals were housed in an environmentally controlled, pathogen-free animal room with standard conditions: ambient temperature 21°C–24°C, relative humidity 45%–65%, and an artificial light/dark cycle (12/12 h, lights on at 6:00 a.m.). Filtered tap water and nutritionally-balanced rodent chow (Murigran, Agropol S.J., Motycz, Poland) were freely available. The treatment was begun 10 days after the animals’ acclimatization to the laboratory conditions.

Housing and *in vivo* procedures were in strict compliance with Directive 2010/63/EU of the European Parliament and of the Council of 22 September 2010 and the Polish Act of 15 January 2015 on the protection of animals used for scientific or educational purposes. The experimental protocol was approved by the Local Ethics Committee in Lublin (license no. 56/2018).

### 2.2 Treatment and study design

Apigenin (purity >97.0%, purchased from Carbosynth, Compton, UK) was dissolved in 100% dimethyl sulfoxide (DMSO, ICN Biomedicals, Inc., Aurora, OH, United States) and diluted with 0.9% NaCl to reach a final DMSO concentration of 1%. The animals were divided into two groups (n = 10/group): APG-administered 50 mg/kg of apigenin and CONT–control one. To ensure the clarity and reliability of the research, the control group was administered the same vehicle (1% DMSO). Both groups received a single dose every 24 h for 14 days. The doses of apigenin were consistent with those used in comparable *in vivo* studies (Han et al., 2012; Singh et al., 2012; Li et al., 2017; Medhat et al., 2017; Yarim et al., 2022). All solutions were freshly prepared and administered intraperitoneally (i.p.) at a constant volume of 0.1 mL per 10 g of body weight. Apigenin was administered intraperitoneally in order to maximize its bioavailability in the liver (Singh et al., 2012).

### 2.3 Blood analysis, body weight, and liver weight

During decapitation, blood samples were collected into heparinized tubes for analysis. Haematological analysis was carried out about 1 h after sampling using the fully automated ABACUS Junior Vet cell counter haematology analyser (Diatron, Vienna, Austria). Whole blood was analysed for counts of red blood cells (RBC), leukocytes (WBC), lymphocytes (LYM), mid-size leukocytes (MID), and granulocytes (GRA) and percentages of lymphocytes (LY%), mid-size leukocytes (MI%), and granulocytes (GRA%), as well as haematocrit (HCT), haemoglobin (HGB), mean corpuscular volume (MCV), mean corpuscular haemoglobin (MCH), mean corpuscular haemoglobin concentration (MCHC), red blood cell distribution width (RDWc), platelet (PLT) count, mean platelet volume (MPV), and platelet distribution width (PDWc).

Blood plasma for analysis of biochemical parameters was obtained by centrifuging whole blood at 1,000 × g for 10 min in a laboratory centrifuge (MPW-350R, MPW Medical Instruments, Warsaw, Poland) at 4°C. Plasma without signs of haemolysis was examined within 4 h after sampling, and the content of selected compounds, i.e., total protein, albumin, creatinine, urea, uric acid, and bilirubin, as well as the activity of alkaline phosphatase (ALP), alanine aminotransferase (ALT), and aspartate aminotransferase (AST), were assayed using dedicated kits (BioMaxima, Lublin, Poland) according to the manufacturer’s protocols and with a biochemical analyser Metrolab 2300 GL (Metrolab SA, Buenos Aires, Argentine). Analytical procedures were validated using multiparametric control plasma (BioCal), as well as control plasma with normal (BioNorm) and high (BioPath) levels of blood parameters (BioMaxima, Lublin, Poland; Hydrex Diagnostics, Warsaw, Poland).

The mice were weighed before the start of the experiment and after its completion. The liver of each animal in the control and experimental groups was weighed following its rapid removal from the abdominal cavity.

### 2.4 Tissue collection

One hour after the final administration of apigenin, the animals were euthanized by decapitation. Liver samples were collected from all experimental animals. Tissue samples were not pooled. The liver was quickly removed from the abdominal cavity and washed in ice-cold saline (87 mM NaCl, 2.5 mM KCl, 1.25 mM NaH_2_PO_4_, 25 mM NaHCO_3_, 0.5 mM CaCl_2_, 7 mM MgSO_4_, 25 mM glucose, and 75 mM sucrose; pH 7.4). A tissue fragment was taken from each liver sample (left lateral lobe–transverse) for microscopical examination, and the remaining liver tissues were stored at −80°C until proteomic and cytokine analysis. The sampling methodology for the microscopical examination was described by Fiette and Slaoui (2011).

### 2.5 Microscopical examination of the liver

Liver tissue samples (left lateral lobe–transverse) were fixed for 24 h in 4% buffered formalin, processed in a tissue processor (Leica TP-1020, Biosystems, Germany), and embedded in paraffin (EM-850015, Pathosolutions, Elektro Med, Poland). H&E staining was performed on 5-µm paraffin sections using a rotary microtome (Slee medical CUT 5062, Aquatech, Germany). Stained tissue sections were evaluated using the Nikon Eclipse E600 light microscope (Nikon Instruments Inc., Japan). Photographic documentation was made with a digital camera (Nikon, DS-Fi1, Nikon Instruments Inc., Japan) and image analysis software (OlympuscellSens Version 1.5; Olympus, Tokyo, Japan). Morphological analysis was performed by a pathologist blinded to the experimental protocol.

The histopathological alterations in the tissue sections were established in 10 random high power fields of view and scored from 0 to 3, where 0 = no alteration, 1 = slight alteration, 2 = moderate alteration, and 3 = severe alteration. All procedures have been described by Bancroft and Gamble (2002).

### 2.6 Assay of IL-1β, IL-6, IL-10, IL-18, IFN-γ, TNF-α, and toll-like receptor 4 (TLR-4) in mouse liver samples

ELISA kits were used to determine IL-1β, IL-6, IL-10, IFN-γ, TNF-α, TLR-4 (EIAab Science Inc, Wuhan, China no. E0563m, E0079m, E0056m, E0049m, E0133m, E0753m), and IL-18 (Biorbyt Ltd., Cambridge, United Kingdom, no. orb437211) in the mouse liver tissues (10 samples from the control group and 10 from the experimental group). All assays were performed according to the manufacturer’s instructions. All samples were tested in triplicate. The data obtained were expressed precisely per mg of tissue protein.

### 2.7 Protein extraction

Livers (six samples from the control group and six from the experimental group) were cut into small pieces, washed with 0.9% NaCl, and homogenized in TRIS-HCl (1.5 M in water, pH 8.8). The homogenates were made using a T10 basic IKA homogenizer (Germany). The samples were then purified and concentrated using Amicon Ultra-0.5 3 kDa filters (Merck KGaA, Darmstadt, Germany). Subsequently, 180 µg of protein precipitates were obtained using the Ready-Prep™ 2-D Cleanup Kit (Bio-Rad, Warsaw, Poland). Pellets were dissolved in rehydration buffer (Bio-Rad, Warsaw, Poland). The protein mixtures were poured onto a rehydration plate, and 17-cm immobilized pH gradient (IPG) linear strips for isoelectric focusing were placed on top (ReadyStrip IPG, pH 3–10, Bio-Rad, Warsaw, Poland). The strips were covered with mineral oil (Bio-Rad, Warsaw, Poland) and left to rehydrate for 12 h.

After rehydration, the strips with proteins were placed in a Hoefer IEF100 apparatus (Hoefer, Inc., Holliston, MA, United States) for electrophoretic isofocusing under the following conditions: 250 V for 30 min, 10,000 V for 3 h, and a total of 60 kV/h, with a current limit of 50 μA per strip. Before the second dimension was run, the strips were equilibrated in solutions of 1,4-dithiothreitol and iodoacetamide. The equilibrated strips were used for the second dimension of electrophoresis at 600 V, 30 mA, and 100 W in a PROTEAN® II xi electrophoretic chamber (Bio-Rad, Warsaw, Poland). After electrophoresis, the gels underwent silver staining in the presence of formaldehyde. The stained gels were scanned using the Image Scanner III (GE Healthcare, Warsaw, Poland) and analysed in Delta2D graphic and statistical software (version 4.7, DECODON, Greifswald, Germany). The software was used to align protein spots across all gels, creating a composite protein map containing all observed protein spots. Expression ratios were calculated, and statistical analysis was performed using a t-test (P ≤ 0.05).

### 2.8 Protein identification

Selected proteins were excised from the gels, destained, reduced with dithiothreitol, and alkylated with iodoacetamide solutions. The gel pieces were then digested with trypsin in 50 mM bicarbonate buffer at 37°C for 12 h (Promega, Trypsin Gold, Mass Spectrometry Grade). The resulting peptides were extracted using a water/acetonitrile/TFA solution (450:500:50 v/v) by triple extraction. Peptide mixtures were treated with Zip-tip (Merck Chemicals, Billerica, MA, United States). Prepared peptide and standard solutions (Peptide Calibration Standard II, Bruker, Bremen, Germany) were spotted on an Anchor Chip MALDI plate (Bruker, Bremen, Germany) and covered with 1 μL of α-cyano-4-hydroxycinnamic acid matrix (HCCA, Bruker, Bremen, Germany).

Mass spectra were acquired in positive reflector mode within the 700–4,000 m/z range using an Ultraflextreme MALDI TOF/TOF spectrometer (Bruker, Bremen, Germany) and flexControl 3.3 software (Bruker, Bremen, Germany). The spectra were smoothed and baseline corrected. Peaks with a signal-to-noise ratio greater than three were identified using flexAnalysis 3.0 software (Bruker, Bremen, Germany), and the peak list was transferred to BioTools 3.2 (Bruker, Bremen, Germany) for comparison with the Swiss-Prot database (www.uniprot.org) using Mascot 2.2 software (Matrix Science, Boston, MA, United States). The search was restricted to ‘mus’, with a maximum error of 0.3 Da and cysteine carbamidomethylation as a fixed modification. Results with a Mascot score above 55 were considered significant (p ≤ 0.05). For lower scores, combined ion spectra of selected peptides were obtained using the LIFT mode and subjected to further MALDI TOF/TOF identification.

### 2.9 Statistical analysis

On the basis of spot volumes, differences in protein synthesis between the test groups were analysed by t-test with alpha critical value p ≤ 0.05 based on t-distribution in Delta2D 4.7.0 (DECODON, Greifswald, Germany). A spot intensity ratio higher than 1.3 (upregulated) or lower than 0.67 (downregulated) was the basis for protein identification. The results were presented in Figures 3, 4; Tables 4, 5.

Statistical analyses of cytokine concentrations in the liver samples were performed using Statistica 13.2 software (Stat. Soft, Inc., Krakow, Poland). After the data were tested for normality (Shapiro–Wilk test), the non-parametric Mann–Whitney *U* test was performed to determine whether there was a significant difference between the means of the two groups, control (CONT) and experimental (APG). Results were expressed as mean and standard deviation (±SD), and differences were considered significant at p < 0.05.

The haematological and biochemical blood parameters results in mice were compared by Student’s t-test from SAS system (SAS® v. 9.4 statistics software, 2011). Statistical significance was set at p ≤ 0.05; *p ≤ 0.05; **p ≤ 0.01. NS - no statistically significant. The results are presented in Table 1.

**TABLE 1:** Effects of apigenin on selected haematological and biochemical blood parameters in mice.

Parameter	Control	Apigenin	P Value summary
WBC (10^9^/L)	4.07 (0.98)	4.94 (0.68)	↑*
LYM (10^9^/L)	2.51 (0.71)	2.68 (0.38)	NS
MON (10^9^/L)	0.14 (0.05)	0.15 (0.04)	NS
NEU (10^9^/L)	1.37 (0.31)	2.02 (0.47)	↑**
EOS (10^9^/L)	0.050 (0.008)	0.052 (0.021)	NS
BAS (10^9^/L)	0.035 (0.007)	0.047 (0.014)	↑*
LYM (%)	60.58 (4.08)	54.63 (5.48)	↓**
MON (%)	3.84 (1.75)	3.02 (0.89)	NS
NEU (%)	34.40 (3.45)	40.38 (5.07)	↑**
EOS (%)	1.31 (0.33)	1.01 (0.29)	↓*
BAS (%)	0.96 (0.34)	0.94 (0.22)	NS
RBC (10^12^/L)	10.25 (0.96)	10.91 (0.48)	↑*
HCT (%)	54.00 (4.86)	58.63 (2.35)	↑**
HGB (g/L)	15.33 (1.41)	15.97 (0.79)	NS
MCV (fL)	52.92 (3.63)	53.50 (1.67)	NS
MCH (pg)	14.96 (0.47)	14.58 (0.45)	NS
MCHC (g/L)	28.40 (1.70)	27.25 (0.46)	↓*
RDWc (%)	39.08 (2.27)	44.76 (4.17)	NS
PLT (10^9^/L)	551 (135)	761 (107)	↑*
MPV (fL)	6.98 (0.33)	6.92 (0.30)	↓**
PCT (%)	0.39 (0.10)	0.53 (0.09)	NS
ALT (U/L)	66.8 (14.8)	73.2 (40.4)	NS
AST (U/L)	322 (59.4)	272 (63.6)	NS
Bilirubin (μmol/L)	16.4 (4.48)	11.6 (1.65)	NS
Creatinine (μmol/L)	42.6 (10.5)	46.0 (13.2)	NS
Blood urea nitrogen (mmol/L)	18.5 (5.68)	18.9 (5.85)	NS
Urea (mmol/L)	39.6 (12.8)	40.5 (12.5)	NS
Uric acid (mmol/L)	0.508 (0.034)	0.510 (0.032)	NS
Alkaline phosphatase (U/L)	15.4 (9.45)	14.7 (13.0)	NS
Total protein (g/L)	57.4 (4.25)	52.3 (6.63)	NS
Albumin (g/L)	37.6 (3.83)	33.8 (3.66)	NS

Apigenin (50 mg/kg) was administered *ip* for 14 days. Blood samples were collected 60 min after the last injection. Data are presented as means (SD), n = five to six mice/group. Data were analysed using Student’s t-test. NS, no statistically significant; p ≥ 0.05; *p ≤ 0.05; **p ≤ 0.01. Abbreviations: WBC, number of leukocytes; NEU, number of neutrophils; LYM, number of lymphocytes; MON, number of monocytes; EOS, number of eosinophils; BAS, number of basophils; NEU%, percentage of neutrophils; LYM%, percentage of lymphocytes; MON%, percentage of monocytes; EOS%, percentage of eosinophils; BAS%, percentage of basophils; RBC, number of red blood cells; HCT, haematocrit; HGB, haemoglobin; MCV, mean corpuscular volume; MCH, mean corpuscular haemoglobin; MCHC, mean corpuscular haemoglobin concentration; RDWc, red blood cell distribution width; PLT, platelets; MPV, mean platelet volume; PDWc, platelet distribution width; ALT, alanine aminotransferase; AST, aspartate aminotransferase.

## 3 Results

### 3.1 Blood parameters, body weight, and liver weight

The effects of apigenin treatment on the haematological and biochemical blood parameters in mice are summarized in Table 1. The experimental group had a higher leukocyte count in the blood (4.94 vs. 4.07 10^9^/L, p = 0.02), while numbers of neutrophils (2.02 vs. 1.37 10^9^/L, p = 0.001) and basophils (0.047 vs. 0.035 10^9^/L; p = 0.03) were higher in the apigenin-treated group. Some differences in RBC count were also observed between the groups. Apigenin-treated mice had significantly higher RBC counts and haematocrit values than the control group, but there was no effect on other erythrocyte parameters–HGB, MCHC, MCV, MCH, or RDW. Interestingly, PLT count and MPV were also affected by apigenin treatment, with a more than 30% increase in PLT count in the experimental group. Biochemical analysis of the blood plasma showed no significant changes in the markers analysed. The lack of differences in enzyme activity (ALT, AST, and ALP) and unaltered values ​​of other markers (e.g., total protein, albumin, bilirubin, urea, uric acid, and creatinine levels) demonstrate that the treatment had no negative effects on liver function. See Table 1.

The initial and final body weights (g) of mice in the control group were 29.10 ± 2.51 and 31.40 ± 3.60, respectively. The initial and final body weights of mice in the apigenin supplemented group were 27.90 ± 3.14 and 31.20 ± 3.55, respectively. The absolute (g) and relative (liver weight/100 g body weight) liver weights of mice in the control group were 1.577 ± 0.181 and 5.02 ± 0.08, respectively. The absolute (g) and relative (liver weight/100 g body weight) liver weights of mice in the apigenin supplemented group were 1.528 ± 0.174 and 4.90 ± 0.011, respectively. No statistically significant differences in initial and final body weight as well as absolute or relative liver weight were observed between the control group and the apigenin-supplemented group.

### 3.2 Microscopical examination results

**FIGURE 1:**
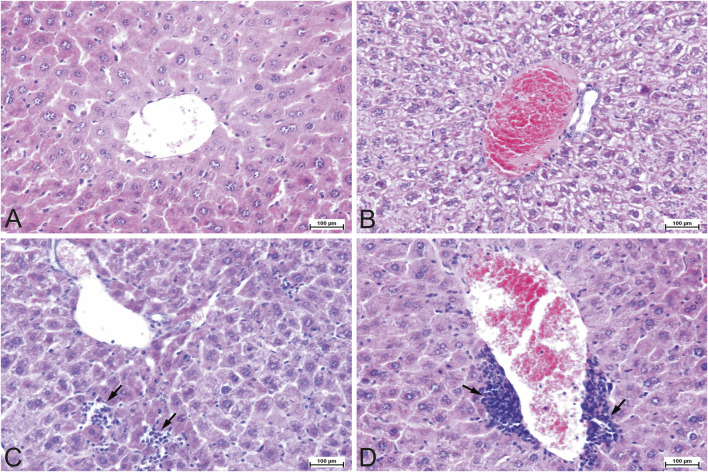
Representative photomicrographs of the mouse liver tissue, H&E staining, ×200 magnification. **(A)** Control (CONT) group. Tissue section of a normal liver with the central vein and hepatocyte cords. **(B)** Experimental (APG) group. Congested and engorged central blood vessel with moderate to severe hydropic degeneration in the parenchyma. **(C)** Experimental (APG) group. Interstitial inflammatory lymphocytic infiltrates (arrows). **(D)** Experimental (APG) group. Perivascular mononuclear inflammatory infiltrates (arrows). APG–apigenin.

**FIGURE 2:**
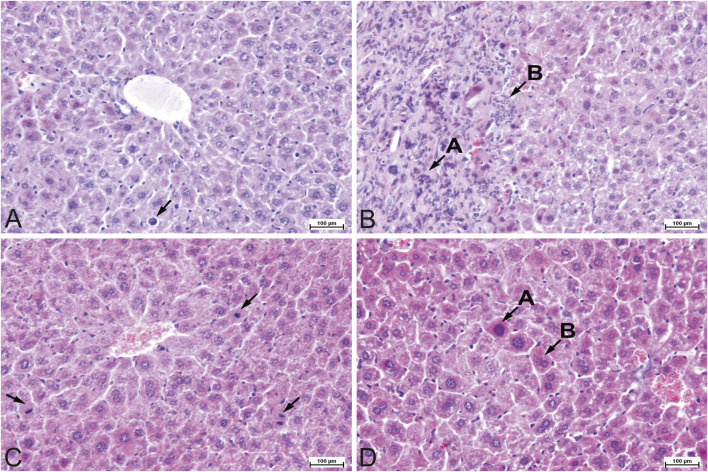
Representative photomicrographs of the mouse liver tissue, H&E staining, ×200 magnification. **(A)** Experimental (APG) group. Apoptosis of individual hepatocyte (arrow). **(B)** Experimental (APG) group. Interstitial fibrosis **(A)** with neutrophilic inflammatory infiltrates **(B)**. Slight dissociation of hepatocytes with distortion of the liver architecture. **(C)** Experimental (APG) group. Mitotic figures (arrows) in hepatocytes as a sign of proliferation of individual hepatocytes. **(D)** Experimental (APG) group. Karyomegaly **(A)** with binucleation **(B)** in individual hepatocytes. Moderate dissociation of hepatocytes with distortion of the liver architecture.

A slight degree of parenchymal degeneration (hydropic change) was observed in most (8/10) mice in APG group and moderate parenchymal degeneration was observed in single cases in both groups (Figure 1B). A few individuals (3/10) in the control group showed a slight degree of karyomegaly, as did half of the apigenin-exposed mice, and one individual in APG group showed a moderate degree of karyomegaly (Figure 2D). In most (6/10) mice exposed to apigenin, a slight degree of hepatocyte dissociation was observed as well (Figure 2B), and in one case a moderate degree of hepatocyte dissociation (Figure 2D). In the apigenin-exposed group, 2/10 individuals showed binucleated hepatocytes (Figure 2D) and in one individual a single focus of perivascular inflammatory infiltration was found (Figure 1D). Other minor changes were observed incidentally in APG group, such as focal activated fibroblasts, focal inflammatory infiltrates effacing the parenchyma (Figure 1C;Figure 2B), congestion (Figure 1B), individual apoptosis (Figure 2A), and single mitotic figures (Figure 2C). Evaluation of APG group revealed slight to moderate parenchymal degeneration, slight to moderate dissociation of hepatocytes, slight activated hepatocytes, and the above-mentioned morphological changes were probably test item-related. Other changes like interstitial inflammatory infiltrates and congestion were incidental and probably not test item-related (Table 2).

**TABLE 2:** Histopathology score of hepatic lesions.

	No.	Rounded edges	Parenchymal degeneration	karyomegaly	Subcapsular, single inflammatory focus (neutrophils)	Stimulated fibroblasts	Infiltrates effacing the parenchyma	Extravasation	Binucleated hepatocytes	Dissociation of hepatocytes	Mitotic figures	Hyperplasia of hepatocytes	Perivascular inflammatory infiltrates composed of neutrophils	Inflammatory infiltrates in the biliary spaces composed of lymphocytes	Small inflammatory infiltrates composed of mononuclear cells in the biliary spaces	Fusion of the capsule with muscle fibres on the diaphragmatic surface	Inflammatory infiltrates composed of mononuclear cells in the subcapsular area, focally adjacent to the diaphragm	Hyperaemia	Engorged blood vessels	Blood clots	Bile duct epithelial hyperplasia	Single focus of eosinophilia infiltration adjacent to a vein	Neutrophilic infiltrates in the peripheral zone with reactive stroma	Small foci of inflammatory infiltrates composed of lymphocytes in the vicinity of veins	Single interstitial infiltrates composed of eosinophilia
**CONT**	1		1	1																					
	2																								
	3																								
	4																								
	5			1																					
	6																								
	7									1															1
	8																								
	9		1																			1			
	10			1																					
**APG**	11	1	1																						
	12	2	1	2	1	1	1	1	2	1	1	1	1	1											
	13	1	1							1					1	1	1								
	14	1	2	2					1									1	1	1					
	15	1	1							1					1						1				
	16		1	2						1					1					1		1			
	17	1								1													1	1	
	18		1																						
	19	1		2						2		2						1							1
	20	1	1	1						1												1			

CONT, control group; APG, apigenin, experimental group. 1 — slight alteration, 2 — moderate alteration, 3 — severe alteration, No. – mouse sample number.

### 3.3 Concentrations of cytokines IL-1β, IL-6, IL-10, IL-18, IFN-γ and TNF-α and of toll-like receptor 4 (TLR-4) in the liver of mice

No statistically significant differences were observed in the concentrations of cytokines IL-1β, IL-6, IL-10, IL-18, IFN-γ and TNF-α or of toll-like receptor 4 (TLR-4) in the liver of mice between the control group and the group of mice subjected to apigenin (Table 3).

**TABLE 3:** Liver concentrations of IL-1β, IL-6, IL-10, IL-18, IFN-γ, TNF-α and toll-like receptor 4 (TLR-4) in mice from control (CONT) and experimental (APG) groups.

Parameter	CONT	APG	*p-value*
	N = 10	N = 10	
IFN-γ [pg/mg protein]	239.97 ±1.9	244.1 ± 4.8	0.09ns
IL- 1β [pg/mg protein]	22.74 ±0.18	22.76 ±0.15	0.94ns
IL-6 [pg/mg protein]	307.18 ±4.99	309.45 ±9.61	0.69ns
IL-10 [pg/mg protein]	299.72 ±11.41	305.65 ±17.39	0.81ns
IL-18 [ng/mg protein]	161.82 ±9.85	163.16 ±10.16	0.94ns
TLR-4 [ng/mg protein]	10.84 ±0.53	11.18 ±0.14	0.07ns
TNF-α [pg/mg protein]	160.67 ±3.67	161.21 ±1.84	0.81ns

The Mann–Whitney *U*test was used to show the significance of statistical differences (p < 0.05) between the control (CONT) and experimental group (APG). Values are expressed as mean and standard deviation (±SD). APG, apigenin, N–number of samples, and p-values indicating that there were no statistically significant (ns) differences between the control and experimental groups.

### 3.4 Protein identification

**FIGURE 3:**
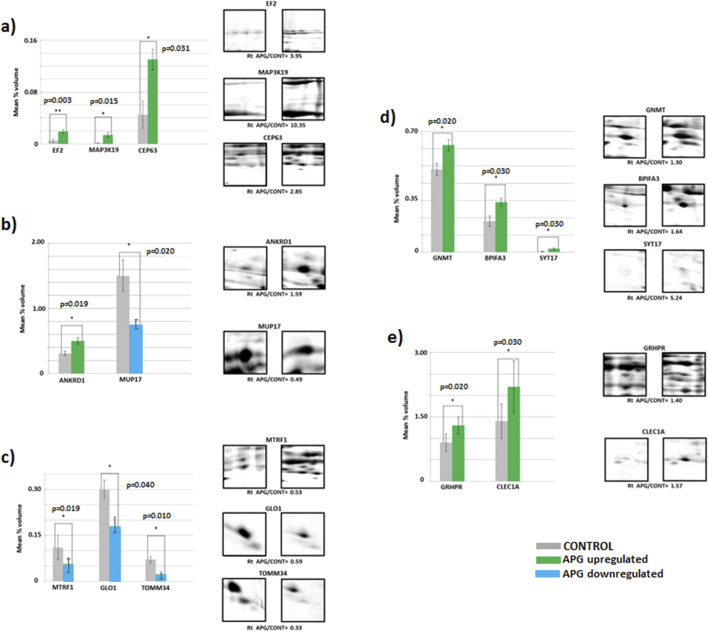
Mean volumes (%) of protein spots in the experimental (APG, six gels) and control (CONT, six gels) groups. Statistically different proteins are compared, and ratio parameters (Rt) are given. **(a)** EF2 – elongation factor 2, MAP3K19 – mitogen-activated protein kinase kinase kinase 19, CEP63 – centrosomal protein of 63 kDa; **(b)** ANKRD1 – ankyrin repeat domain-containing protein 1, MUP17 – major urinary protein 17; **(c)** MTRF1 – peptide chain release factor 1, mitochondrial, GLO1 – lactoylglutathione lyase, TOMM34 – mitochondrial import receptor subunit TOM34; **(d)** GNMT–glycine N-methyltransferase, BPIFA3 – BPI fold-containing family A member 3, SYT17 – synaptotagmin-17; **(e)** GRHPR–glyoxylate reductase/hydroxypyruvate reductase, CLEC1A–C-type lectin domain family one member **(a)**. Significant differences assessed using a t-test are marked with asterisks: *P ≤ 0.05, **P ≤ 0.01.

**FIGURE 4:**
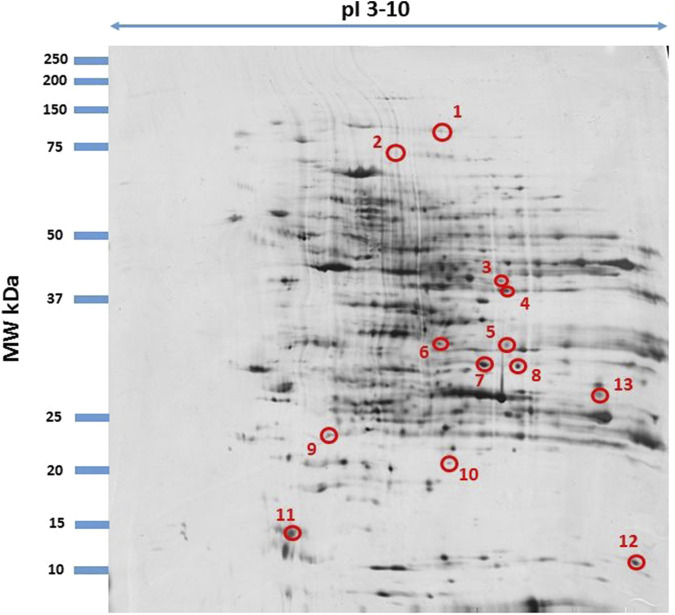
Fused image showing condensed spot patterns from the experiment. The differentially expressed proteins are marked with circles. Proteins were separated in the first dimension by isoelectric focusing over the isoelectric point (pI) range 3–10. The second dimension was performed using a 12.5% sodium dodecyl sulphate polyacrylamide gel. Gels were silver stained, digitized, and processed in Delta2D software (version 4.7 DECODON Greifswald, Germany).

Among the proteins analyzed in the samples, we focused only on those with differential electrophoretic spots. MALDITOF mass spectrometry identified 13 statistically significant proteins (Table 4). Table 4 contains a list of the protein names, UniProt base accession numbers, and ANOVA P values. Stains were positively identified as elongation factor 2 (EF2); mitogen-activated protein kinase kinase kinase 19 (MAP3K19); centrosomal protein of 63 kDa (CEP63); peptide chain release factor 1, mitochondrial (MTRF1); ankyrin repeat domain-containing protein 1 (ANKRD1); the enzyme glyoxylate reductase/hydroxypyruvate reductase (GRHPR); the enzyme glycine N-methyltransferase (GNMT); BPI fold-containing family A member 3 (BPIFA3); C-type lectin domain family one member A (CLEC1A); synaptotagmin-17 (SYT17); major urinary protein 17 (MUP17); the enzyme lactoylglutathione lyase (GLO1); and mitochondrial import receptor subunit TOM34 (TOMM34). Table 5 contains a list of the protein names, UniProt base accession numbers, and ANOVA P values. See also Table 5, which provides an overview of the functions of the identified proteins. According to the results obtained with Delta2D software, the nine proteins were classified as upregulated (Figure 3). Figure 4 shows a fused image of 2D gels with differentially expressed proteins in one of the experimental groups *versus* the control group.

**TABLE 4:** Differentially expressed proteins in mouse livers identified by MALDI-TOF MS.

Id	Protein	Accession number (UniProtKB)	Score	Match	MW (Da)*	pI*	Modif	Seq. Cov(%)	t-test p value	Rt
1	Elongation factor 2	O89070	174	15	96222	6.41	C, O	20	0.003	3.95
2	Mitogen-activated protein kinase kinase kinase 19	E9Q3S4	100	10	147916	5.85	C	6	0.015	10.35
3	Peptide chain release factor 1, mitochondrial	Q8K126	177	14	53052	8.63	C	18	0.031	0.53
4	Centrosomal protein of 63 kDa	Q3UPP8	140	10	80855	5.52	C, O	21	0.015	2.85
5	Ankyrin repeat domain-containing protein 1	Q9CR42	93	6	36223	8.81	C, O	16	0.019	1.59
6	Glyoxylate reductase/hydroxypyruvate reductase	Q91Z53	150	9	35706	7.57	C, O	32	0.02	1.40
7	Glycine N-methyltransferase	Q9QXF8	85	11	33111	7.10	C	59	0.02	1.30
8	BPI fold-containing family A member 3	Q9D9J8	119	7	25982	8.87	C, O	18	0.03	1.64
9	C-type lectin domain family 1 member A	Q8BWY2	96	6	31565	7.42	C, O	13	0.03	1.57
10	Synaptotagmin-17	Q920M7	140	11	53887	6.71	C, O	11	0.03	5.24
11	Major urinary protein 17	B5X0G2	102	13	20921	4.89	C, O	71	0.02	0.49
12	Lactoylglutathione lyase	Q9CPU0	106	6	20967	5.24	C, O	22	0.04	0.59
13	Mitochondrial import receptor subunit TOM34	Q9CYG7	85	6	34656	9.24	C, O	12	0.01	0.33

Abbreviations: C–carbamidomethylation of cysteine, O–oxidation of methionine, Rt–ratio parameters, Match–number of overlapping peptides in the database, Score–protein score is −10*Log(P), where P is the probability that the observed match is a random event. Protein scores greater than 55 are significant (p < 0.05). Listed molecular weights and pI values correspond to the MASCOT, Search Result. Significantly differences (p < 0.05) were assessed by t-test.

**TABLE 5:** Key functions of differentially expressed proteins in the liver of mice after 2-week treatment with apigenin.

Protein name	Abbreviated form	Key role	References
Elongation factor 2	EF2	Plays essential role in protein synthesis. A GTP-binding protein mediating the translocation of peptidyl-tRNA from the A site to the P site of the ribosome	Kaul et al. (2011)
Mitogen-activated protein kinase kinase kinase 19	MAP3K19	Modulates ERK and JNK signalling cascades; regulates NF-κB to promote the release of cytokines	Boehme et al., 2016 Hoang et al. (2020)
Peptide chain release factor 1, mitochondrial	MTRF1	A mitochondrial translation factor that directs translation termination at non-canonical stop codons (AGG and AGA)	Krüger et al., 2023
Centrosomal protein of 63 kDa	CEP63	A constitutive centrosomal protein involved in spindle assembly and spindle inactivation following DNA damage; regulates mother-centriole-dependent centriole duplication and recruits Cdk1 to the centrosome	Löffler et al., 2011 Wu et al. (2021)
Ankyrin repeat domain-containing protein 1	ANKRD1	May participate in various cellular processes (e.g., transcriptional regulation); presumed to be involved in liver fibrosis	Ghaffari et al. (2024)
Glyoxylate reductase/hydroxypyruvate reductase	GRHPR	An enzyme that catalyses the reduction of hydroxypyruvate to D-glycerate and of glyoxylate to glycolate, as well as oxidation of D-glycerate to hydroxypyruvate; plays a crucial role in the removal of the metabolic by-product glyoxylate from the liver	Rumsby and Cregeen, 1999 Booth et al. (2006)
Glycine N-methyltransferase	GNMT	Catalyses the transfer of a methyl group from SAM to glycine to form SAH and N-methylglycine (sarcosine); plays a significant role in preserving the physiologically normal SAM/SAH ratio, which is necessary for controlling the methylation potential within the cell	Pakhomova et al. (2004)
BPI fold-containing family A member 3	BPIFA3	Presumed to be a lipid-binding protein playing some role in bacterial host defence, but its exact function is unknown	Rye et al. (2012)
C-type lectin domain family 1 member A	CLEC1A	A member of the CLR receptor family having various functions, including the regulation of inflammation; recognizes necrotic cells and reduces the acute inflammatory response after liver injury	Ligeron et al. (2024)
Synaptotagmin-17	SYT17	Belongs to a family of membrane-trafficking proteins involved in vesicular trafficking and exocytosis; the role of SYT17 in the mouse liver is unknown	Wolfes and Dean (2020)
Major urinary protein 17	MUP17	Belongs to a family of proteins that bind volatile pheromones and other lipophilic compounds; they are involved in pheromone transportation, excretion in the kidney, and release into the air from urine marks	Zhou and Rui (2010)
Lactoylglutathione lyase	GLO1	An important component of the glyoxalase system that catalyses the conversion of reactive acyclic α-oxoaldehydes to α-hydroxyacids; plays a crucial role in the detoxification of cytotoxic methylglyoxal	Thornalley (2003)
Mitochondrial import receptor subunit TOM34	TOMM34	A co-chaperone that participates with chaperones HSP70 and HSP90 in mitochondrial precursor protein transport in the cytosol	Faou and Hoogenraad (2012)

Abbreviations: ERC, extracellular signal–regulated kinase; HSP70 — heat shock protein 70 kDa; HSP90 — heat shock protein 90 kDa; JNK—JUN N-terminal kinase; SAH—S-adenosylhomocysteine; SAM—S-adenosylmethionine.

### 3.5 Protein profile of liver tissue

The results indicate significantly higher expression of proteins MAP2K19, CEP69, GNMT, BPIFA3, SYT17, ANKRD1, GRHPR, CLEC1A and EF2 (p ≤ 0.001) in the livers of mice in the group subjected to apigenin compared to the control group (Figures 3a–e). In the case of proteins MUP17, MTRF1, GLO1 and TOMM34, significantly higher expression (p ≤ 0.05) was observed in the livers of mice from the control group compared to the experimental group (Figures 3a–e).

## 4 Discussion

The molecular mechanisms underlying the effects of apigenin on cells and their metabolism have not been fully explained. Published research results on this subject mainly concern models of acute exposure of animals to various doses of apigenin administered by various routes. For example, Singh et al. (2012) showed that single intraperitoneal administration of apigenin to mice at doses of 25, 50, 100 and 200 mg/kg led to the activation of 48 genes involved in various biological processes, e.g., in the regulation of apoptosis, stress, and cell growth and in transcription and translation. The present study provides the first evidence of changes in the proteomic profile of the liver of mice in response to subacute exposure to apigenin. We identified 13 proteins, performing numerous biological functions, whose profile made it possible to distinguish between the experimental and control groups.

One change in expression was shown for the protein Clec1A, which is one of the receptors (CLR) involved in defending the host against pathogens (Thebault et al., 2009). High Clec1a mRNA expression has been observed in the lungs, lymph nodes, spleen, and aorta of rats (Thebault et al., 2009) and in the cells of the lungs, liver, heart, kidneys, and small intestine of mice (Stappers et al., 2018). Our experiment showed expression of Clec1A in the liver of mice receiving apigenin. Literature data also indicate that Clec1A stimulates a pro-inflammatory response, e.g., in the nervous system, leading to degenerative changes (Thebault et al., 2009). In the present study, exposure to apigenin did not induce changes in the concentrations of pro- and anti-inflammatory cytokines in the liver between the experimental and control groups. However, high concentrations of pro-inflammatory cytokines were observed in the liver tissue and morphological changes were demonstrated, including inflammatory infiltrates. The results of the study indicate that the increased expression of Clec1A can be linked to the inflammatory changes in the liver induced by the administration of apigenin. It is worth noting that if this protein is present in excessive amounts, it can contribute to the progression of neoplastic changes by promoting the immunosuppressive tumour microenvironment (TME) and disturbing tumour antigen presentation to CD8^+^ T lymphocytes by dendritic cells (Drouin et al., 2022). The morphological changes observed in the liver as a result of supplementation with apigenin may suggest that over a longer period of time, neoplastic processes might have developed. On the other hand, Clec-1A is able to recognize necrotic cells and limit the acute inflammatory response, e.g., resulting from trauma (Ligeron et al., 2024). Therefore, the increase in the expression of this protein following administration of apigenin in the experiment may also be linked to the limitation of inflammatory changes in the liver, which is supported by the high concentration of anti-inflammatory Il-10. Given the specific properties of apigenin, expressed as a reduction in liver damage through mitigation of inflammation and oxidative stress, its use to treat liver inflammation is justified, e.g., in the case of HCV infections (Shibata et al., 2014; Yue et al., 2020).

In our experiment, mice receiving apigenin showed higher expression of glyoxylate reductase/hydroxypyruvate reductase (GRHPR) in the liver tissue than the controls. These results confirm previous findings regarding the effect of apigenin on glucose metabolism, the activity of gluconeogenic enzymes, increased glucose tolerance, and other processes associated with gluconeogenesis (Ohno et al., 2013; Bumke-Vogt et al., 2014). It can also be assumed that the increased energy demand of liver cells, expressed by the increased expression of this protein, will be associated with the processes of regeneration of damaged hepatocytes and reconstruction of the organ structure.

The results of the present study demonstrate that exposure to apigenin causes an increase in the expression of protein kinase MAP3K19 in the liver of mice. Mitogen-activated protein kinases (MAPKs) are a group of serine/threonine protein kinases which play an important role in regulating the cellular response to external signals and take part in gene expression, cell division and differentiation, and apoptosis (Roux and Blenis, 2004). The increased expression of this protein in the present study may not be related to any pathological process in the hepatocytes, including carcinogenesis, during which a decrease in MAP3K19 expression would be expected. Similar results were obtained by Nguyen et al. (2022). It seems likely that hepatocytes were stimulated by apigenin during our experiment, and metabolic processes and cell differentiation and development were activated. The activation of such processes may also be evidenced by high concentrations of cytokines with immunoregulatory properties, including Il-6, Il-10, and TNF-α. Mitogen-activated protein kinases (MAPKs) are additionally involved in cellular metabolism (Gehart et al., 2010; Lawan and Bennett, 2017), the development of liver inflammation, and fibrosis (Westenberger et al., 2021). The high expression of MAP3K19 in conjunction with the microscopical changes in the liver in the form of mixed parenchymal inflammatory infiltrates involving predominantly granulocytes suggests that the use of apigenin, on the one hand, through the expression of this protein, may enhance the apoptosis processes of damaged cells and, on the other hand, its long-term use could adversely affect the body, leading to inflammation and, secondarily, to fibrosis of the liver.

In our experiment, mice receiving apigenin also showed high expression of elongation factor 2 (EF2) and centrosomal protein 63 (CEP63) in the liver. CEP63, together with other proteins of the CEP family, is located in the centrosome (Kumar et al., 2013) and plays an important role in the cell cycle (Einarsdottir et al., 2015). The high expression of this protein shown in the liver may be linked to the proliferation of hepatocytes during the growth and development of the liver, as well as to the regeneration of liver cells, in which inflammatory foci resulting from the use of apigenin are visible. EF2, on the other hand, is involved not only in the synthesis of cellular proteins, but also in the organization of the mitotic apparatus, signal transmission, and regulation of cell development, ageing, and transformation (Riis et al., 1990). The results of the present study therefore indicate that EF2, like CEP63, takes part in the differentiation and proliferation of hepatocytes under the influence of apigenin. Our hypothesis that application of apigenin stimulates cell differentiation, division, and regeneration is confirmed by Singh et al. (2012), who administered apigenin intraperitoneally to mice at various doses and observed an increase in the expression of genes involved in the transcription and translation of proteins regulating cell growth. It should be noted, however, that the effects of the use of apigenin on the body are not fully known, and it may also inhibit cellular metabolism and have cytotoxic effects (Khandelwal et al., 2020).

The results of the present study also indicate that administration of apigenin to mice is followed by an increase in the expression of the GNMT (glycine N-methyltransferase) protein, which plays an important regulatory role for liver cells by taking part in the synthesis of homocysteine, regulation of metabolism of methyl groups, DNA methylation, biosynthesis of nucleotides, and regulation of genes associated with detoxification and antioxidant pathways (Simile et al., 2018). A deficiency of this protein in mice has been shown to cause steatohepatitis, hepatic fibrosis, and cirrhosis, as well as hepatocellular carcinoma (HCC) (Martinez-Chantar et al., 2008). The results of the present study in conjunction with the histopathological evaluation of the liver of mice demonstrate that apigenin does not lead to the development of neoplasia; in fact, the risk of neoplasia is reduced by high expression of GNMT (Kang et al., 2018; Wang et al., 2021). The effects of apigenin on processes regulating DNA synthesis and cell proliferation conclusively prove that its application promotes the regeneration of damaged liver cells. Rual et al. (Rual et al., 2005) showed that GNMT interacts with other proteins, e.g., those involved in the regulation of MAPK-coupled receptors. In the present study, mice receiving apigenin showed increased expression of MAP3K19 and GNMT, which suggests that the interaction of the two proteins in liver cells, despite histological changes in the liver parenchyma, ultimately probably prevents the development of pathological processes. However, this requires studies with long-term use of apigenin.

Interesting results were obtained regarding the expression of the protein synaptotagmin-17 (SYT17), which was significantly higher in the mice receiving apigenin than in the controls. Synaptotagmins are proteins regulating exocytosis in the cell membrane (Wolfes and Dean, 2020). Among the 17 isoforms of these proteins, SYT17 is the least known. High expression of this protein has been shown in the nervous cells of the brain, mainly in the hippocampus, whereas small amounts can be found in the kidneys. The exact function of SYT17 is unknown, but its increased expression has been shown in cases of ischaemia and seizures (Ruhl et al., 2019). Our experiment showed, for the first time, SYT17 expression in liver cells as a possible consequence of exposure to apigenin. In our opinion, as in the case of the nervous system, SYT17 also takes part in the transport of substances within intracellular membranes in the liver cells, supporting the functional and morphological changes in hepatocytes under the action of apigenin, resulting in slight interstitial inflammatory infiltrates.

Flavonoids, which include apigenin, exert a positive impact on human health, in part through their antioxidant, anti-inflammatory, vasoprotective, antispasmodic (spasmolytic), and diuretic effects (Ullah et al., 2020). Findings recently published by Xu et al. (2013) showed that various subclasses of flavonoids promote the expression of numerous synaptic proteins, including synaptotagmin, in the cortical neurons and hippocampus of rats. This can be exploited in the treatment of neurodegenerative diseases, including Alzheimer’s disease and depression. These findings are confirmed in the present study, in which the administration of apigenin to mice increased the expression of SYT17 in the liver. Flavonoids have also been shown to modulate the formation of synapses and to influence their number and presynaptic activity between cells, which suggests that they influence intracellular signalling processes (Matias et al., 2017). It seems that this type of activity of apigenin can also affect cells of the liver, in which the flavonoid promotes the remodelling and regeneration of hepatocytes, despite the fact that histopathological changes indicate disturbances of hepatocyte morphology and function.

Analysis of the results of our experiment showed that apigenin in mice also causes an increase in hepatic expression of ankyrin repeat domain-containing protein 1 (ANKRD1), also known as cardiac ankyrin repeat protein (CARP), and BPI fold-containing family A member 3 (BPIFA3). ANKRD1 is a nuclear protein induced in the endothelial cells by cytokines, including IL-1 and TNF-α, and its highest expression has been shown in the heart (Miller et al., 2003). Expression of this protein in the liver is low, but it increases in the case of liver disease, e.g., HCV (Pontén et al., 2008). It is worth noting that ANKRD1 expression is also increased by stress (Chu et al., 1995), which is conducive to viral infections of the liver. The use of apigenin as in our experiment may also induce stress in the body, including cells of the liver, in which the flavonoid is metabolized. Increased ANKRD1 expression determined by stress factors should primarily be linked to liver damage and local foci of inflammation. Moreover, high concentrations of IL-1β and TNF-α in the liver are correlated with increased expression of this protein, as well as changes in hepatocyte structures shown in histopathological examination. It should also be emphasized that stress generates an increase in the synthesis of proinflammatory cytokines, and such a mechanism should also be taken into account when considering the use of apigenin.

Analysis of the results of this study indicates increased expression of the BPIFA3 protein in the liver after apigenin administration, which is a member of the BPI-fold protein superfamily and its function is not fully understood. Literature data indicate that the expression of this protein has been demonstrated mainly in the male reproductive organs (testes), but BPIF genes can also encode proteins involved in the recognition of bacterial pathogens in the oral cavity, nasopharynx and lungs (Bingle and Craven, 2002). Increased expression of BPI-fold protein family proteins should be partially associated with inflammation and fibrosis accompanying nonalcoholic fatty liver disease (NAFLD) (Sanyal et al., 2023). The increased BPIFA3 protein concentration we observed may be associated with apigenin-induced inflammation, visible in histopathological preparations of the liver. At the same time, the concentrations of pro- and anti-inflammatory cytokines shown in the study do not confirm the development of viral and bacterial infections, and therefore inflammatory changes in liver cells may be self-limiting and regenerative. Analysis of the results of the experiment should take into account the identification of four proteins whose expression was lower than in the mice receiving apigenin in their diet than in the control group. These include major urinary protein 17 (MUP17), peptide chain release factor 1, mitochondrial (MTRF1), lactoylglutathione lyase (GLO1), and mitochondrial import receptor subunit (TOMM34). MUPs, which belong to the lipocalin superfamily, are expressed mainly in the liver, from which they are released into the bloodstream (Zhou and Rui, 2010). They play an important role in regulating nutrient metabolism and inhibit gluconeogenesis and lipogenesis in the liver (Zhou et al., 2009). The available literature indicates that reduced expression of Mup and lower plasma concentrations of Mup are observed in obese or diabetic mice and in conditions of lower demand for metabolic energy (Zhou and Rui, 2010; Zhou et al., 2009). In contrast, in our study this relationship may have been a manifestation of increased gluconeogenesis and increased production of energy for the metabolism of apigenin and regeneration of hepatocytes. MTRF1, on the other hand, plays an important role in translation processes in the mitochondria, taking part in the synthesis of mitochondrial proteins of importance for respiratory functions and energy production in cells (Nadler and Richter-Dennerlein, 2023). Supplementation with apigenin led to a decrease in the expression of MTRF1 in the liver of mice, which may be linked to its effect on the regulation of translation processes and oxidative stress in the mitochondria. Although the function of MTRF1 is not fully known, it should be assumed that in conditions of increased energy production or in response to oxidative stress, MTRF1 expression in cells may increase in order to ensure homeostasis in the body. Apigenin may lower MTRF1 concentrations despite the pathological changes visible in the liver cells. A reduced MTRF1 concentration accompanied by degenerative processes in the hepatocytes may be associated with apigenin-induced improvement in translation-regulating functions in the mitochondria and a reduction in oxidative stress. An understanding of these associations, however, requires further research on cellular metabolism.

GLO1 is an enzyme which takes part in detoxification processes in cells and protects them against damage caused by reactive aldehydes in conditions of metabolic stress (Berner et al., 2012; Rabbani and Thornalley, 2015). Due to its strong antioxidant properties, apigenin can reduce the severity of oxidative stress through other cellular pathways, e.g., through receptors or proteins other than GLO1, which can lead to a reduction in the expression of GLOI, as seen in our experiment. On the other hand, a low GLO1 level may be linked to cell damage and degeneration processes, observed in neurodegenerative diseases (Vicente Miranda et al., 2016). Administration of apigenin may therefore eventually lead to complete impairment of hepatocyte functions and cause metabolic liver diseases.

The chaperone protein TOMM34 studied in the experiment is essential to normal mitochondrial function due to its involvement in the import of proteins to the mitochondria, as well as in energy production and the regulation of metabolic processes (Heinemeyer et al., 2019; Trcka et al., 2020). Increased synthesis of this protein is observed in conditions of rapid growth and proliferation of cancer cells, in neurodegenerative processes, and in conditions of oxidative stress (Muller et al., 2019). The reduced expression of TOMM34 in the liver of mice receiving apigenin may influence homeostasis of the mitochondria, disturbing their functions, including ATP production. In this way, by inhibiting intensive mitochondrial metabolism, apigenin limits the growth and proliferation of pathological cells, thereby preventing the development of pathological changes such as neoplastic transformation and chronic inflammation. In addition, our study showed that apigenin can modulate the function of other proteins and stimulate signalling pathways, compensating for the reduced expression of TOMM34 in the liver and supporting cellular homeostasis.

**FIGURE 5:**
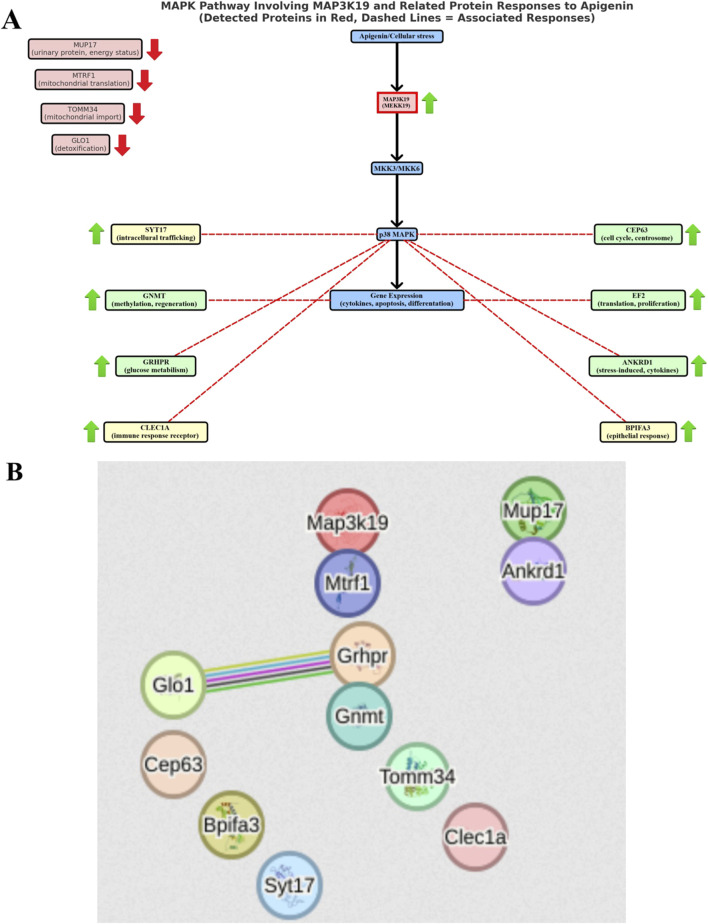
Diagram of probable interactions between proteins synthesized in mouse liver after apigenin administration. **(A)** MAPK (mitogen-activated protein kinases) signaling cascade involving the MAP3K19 protein, **(B)** relationships between liver proteins obtained using STRING software version 12 (STRING: functional protein association networks. Version 12.0. https://string-db.org).

The overall proteomic analysis of our studies revealed the existence of possible interactions between proteins, for which an increase in expression was demonstrated. Using the STRING version 12 software (STRING: functional protein association networks. Version 12.0. Available at: https://string-db.org), we showed that the main metabolic connections occurred between the proteins: lactoylglutathione lyase (GLO1) and glyoxylate reductase/hydroxypyruvate reductase (GRHPR). Interaction analysis confirmed the coexpression of these two proteins (Figure 5B), which may indicate their participation in the same metabolic processes or functional interdependence. According to the KEGG pathway, there is a single interaction record in pyruvate metabolism. According to Navarro et al., 2020, glyoxylate reductase/hydroxypyruvate reductase is an interacting partner of lactoylglutathione lyase in the methylglyoxal detoxification pathway. It is worth emphasizing that increased activity of GLO1, which participates in the processes of methylglyoxal metabolism, was observed in cells subjected to oxidative stress (Thornalley, 2003; Yadav et al., 2005), and its deficiency disrupts this metabolism, which causes toxic effects of methylglyoxal leading to inhibition of cell proliferation, abnormal interactions between proteins and nucleic acids, carcinogenic and mutagenic processes. Therefore, high expression of this protein in the liver demonstrated in our experiment may be associated with resistance to oxidative stress after the use of apigenin, and consequently efficient protection of cells in the metabolic processes of carbon and nitrogen. Special attention should also be paid to the classic MAPK (mitogen-activated protein kinases) signaling cascade, initiated by the activation of the MAP3K19 (MEKK19) protein, in relation to which we demonstrated an increase in expression after the use of apigenin (Figure 5A). Activated MAP3K19 phosphorylates and activates MKK3/6 (MAP kinase kinase 3/6), which in turn activate p38 MAPK (p38 mitogen-activated protein kinases). Active p38 regulates the expression of genes involved in stress response, inflammation, apoptosis, and cell differentiation. As a result of activation of the MAPK pathway, there may be a second stimulation of various metabolic processes and pathways in which liver proteins are functionally involved, in relation to which we have shown an increase in expression after the use of apigenin.

Our experiment did not show differences in the concentrations of pro- and anti-inflammatory cytokines in the liver of mice between the control group and the experimental group receiving apigenin. However, it should be noted that we investigated the effect of apigenien in naïve healthy mice, which can explain the lack of significant influence on inflammatory markers. These findings, despite changes in the hepatocytes in the form of slight inflammatory infiltrates in mice treated with apigenin in the experimental group and with DMSO in the control group, confirmed by the microscopical examination, would suggest that apigenin, in contrast to numerous published research results, does not exert an anti-inflammatory effect at a dose of 50 mg/kg BW. In-depth analysis of cytokine concentrations in the liver in both groups shows that the values obtained in the present study were higher than those normally occurring in mice (Wang N. et al., 2021). Higher values were obtained mainly in the control group, in which DMSO was administered intraperitoneally. It should be noted that this compound has a toxic effect on NKT and NK cells and increases levels of pro-inflammatory cytokines such as IFN-γ (Masson et al., 2008). In the present study, the IFN-γ level in the liver tissue of the control mice was six times as high as in mice receiving 0.9% NaCl (Wang et al., 2021), indicating that its production is stimulated by DMSO, which can result in liver damage. Similar observations were reported by Alanazi et al. (2023), who showed that by stimulating the production of IFN-γ, DMSO causes liver damage and the development of numerous diseases. At the same time, high IL-1 levels were obtained following treatment with DMSO in the control group. This may be explained by an increase in the production of this protein due to the development of local inflammatory changes in the liver, or, as demonstrated by Xing and Remick (2005), DMSO increases IL-1β production in part through an increase in mRNA expression. It should be noted that our microscopical examination showed changes in individual mice in the control group in the form of moderate parenchymal degeneration, slight karyomegaly, slight dissociation of hepatocytes, or focal mixed interstitial infiltrates, which confirms that DMSO administered intraperitoneally can in the long term lead to the development of local inflammatory foci with recruitment of immunocompetent macrophages and neutrophils, systemic inflammation, and liver failure. At the same time, the results of numerous studies emphasize the positive effects of DMSO in the body (Colucci et al., 2008). Its immunomodulatory effect, both in the case of immune suppression and during the promotion of resistance, is manifested in part as decreased proliferation of CD4 and CD8 T cells and reduced secretion of IFN-γ, TNF-α, and IL-2 (de Abreu Costa et al., 2017; Huang et al., 2020). The disturbed expression of pro-inflammatory cytokines observed in the present study in both the experimental and control groups, i.e., elevated expression in the liver tissue, could result in the development of chronic liver disease, impaired liver regeneration, the development of fatty liver disease, and ultimately failure of the organ. Our study additionally showed high levels of IL-6 and IL-10 in the liver, in both the control group and the experimental group of mice receiving apigenin. IL-6 and IL-10 are cytokines with anti-inflammatory and hepatoprotective roles, e.g., in alcoholic liver disease (ALD) (Scarlata et al., 2024). It should be noted that IL-6 itself acts as a pro-inflammatory cytokine in chronic inflammation, but exhibits anti-inflammatory properties during acute inflammation, and its role in the protection of liver tissue is not fully understood (Scarlata et al., 2024). Research has shown that IL-6 activates enzymatic pathways in order to repair mitochondrial DNA in damaged hepatocytes, promotes the differentiation of Th 17 helper cells and the production of IL-17, and inhibits the production of other pro-inflammatory cytokines (Kawaratani et al., 2017; Scarlata et al., 2024). Similarly, IL-10 limits the inflammatory response, thus reducing the degree of liver damage (Scarlata et al., 2024). It is therefore likely that the high levels of these cytokines shown in the liver tissue in the present study are an indication of their hepatoprotective role. They will exert immunoregulatory effects aimed at maintaining homeostasis in the body. It should also be noted that numerous studies on apigenin indicate that its use at various doses reduces mRNA expression of cytokines such as TNF-α, IL-6, and IL-1β in animals treated with various toxic compounds damaging the parenchymal organs, including the liver (Yue et al., 2020; Lu et al., 2023). These values are usually higher than in the control groups, which may indicate that apigenin regulates cytokine production locally at the cellular level (Yano et al., 2006; Yue et al., 2020; Lu et al., 2023). This suggests the need to conduct further molecular studies and to determine whether rapidly and easily absorbed apigenin, administered intraperitoneally, can in the long term disturb the intracellular cytokine network and have cytotoxic effects.

It should be emphasized that in the presented work, the analysis of pro- and anti-inflammatory cytokine concentrations in liver tissue was performed immediately after the last administration of apigenin. However, the amount of pro- and anti-inflammatory mediators produced by the organism can be regulated at many levels of the cell, including in the processes of gene transcription, mRNA translation and mRNA degradation (Stoecklin and Anderson, 2007). Transcription activation processes and post-transcriptional mechanisms regulating the expression of pro-inflammatory mediators are important in both initiating and propagating the inflammatory response and cell regeneration (Anderson and Kedersha, 2006; 2008). Therefore, it is suggested to extend further studies on the effects of apigenin with molecular studies of transcription and translation processes and gene activation at the cellular level, which would provide an answer in which direction the changes induced by apigenin will be propagated. It is also worth emphasizing that apigenin inhibits the activation of nuclear factor kappa B (NF-κB), thereby preventing the upregulation of proinflammatory genes (Stumpo et al., 2010). The low concentrations of cytokines obtained in our experiment may confirm this hypothesis.

Analysis of the haematological and biochemical parameters of the blood showed no adverse effects on blood marker results that might indicate the toxicity of apigenin at a dose of 50 mg/kg BW. This is in line with the study by Singh et al. (2012), who reported that at doses lower than 50 mg/kg, apigenin did not impair liver function and was not hepatotoxic. Although statistically significant differences were shown between the groups, especially for haematological parameters of the blood, i.e., total counts of leukocytes and their subpopulations, e.g., neutrophils and basophils, as well as some red blood cell parameters (RBC count, HCT, and MCHC), the results were within the reference limits for the species (Loeb and Quimby, 1999; Santos et al., 2016; 80; Silva-Santana et al., 2020).

There were also no significant differences in the body weight of the mice, their liver weight, or the liver weight/body weight ratio between the control group and the experimental group receiving apigenin. The liver weight and the liver weight/body weight ratio in the experimental animals may reflect their functional state during the study, while significant changes in these parameters may indicate the toxic effect of a given compound (Liu et al., 2014). Thus the results of the present study suggest that intraperitoneal administration of apigenin had no effect on feed intake during the growth of mice and had no toxic effect on the liver.

Analysis of the results of the microscopical examination showed that in conditions of exposure to apigenin, a variety of minor changes take place in the liver tissue of mice. These include slight parenchymal degeneration, incidental karyomegaly and binucleation of hepatocytes, slight hepatocyte dissociation, mitotic figures in individual hepatocytes, incidental focal inflammatory infiltrates in the parenchyma. These changes indicate a remodeling of the organ in response to enhanced cell metabolism and proliferation, as well as progressive liver degeneration. It is worth noting that even short-term use of apigenin causes morphological changes in the liver. This has been demonstrated by Singh et al. (2012), who observed minor morphological changes in the structure of liver cells, i.e., slight hydropic changes and degeneration of hepatocytes, following single intraperitoneal administration of apigenin to mice at doses of 25 and 50 mg/kg BW. When apigenin was administered at 100 and 200 mg/kg BW, degeneration of hepatocytes were visible in the form of ballooning and hydropic change (Singh et al., 2012). On the other hand, in a study by Taqa and Sultan (2024), oral administration of apigenin to rats at a dose of 50 mg/kg BW for 7 days caused only an increase in the number of Kupffer cells and binucleated hepatocytes. It is worth noting the opposite results were obtained in a study on rats published by Alamri. (2024). Oral administration of apigenin to rats at 50 mg/kg BW daily for 3 weeks resulted in no changes to the liver structure; hepatocytes were normal and exhibited rounded vesicular nuclei and eosinophilic cytoplasm with basophilic granules, with no differences observed in relation to the control group receiving 0.9% NaCl. It can be concluded from the findings cited that apigenin administered at a dose of 50 mg/kg BW for 3 weeks has a neutral effect on the body, which is in clear contrast with the results of the present study.

Taking into account the results of our experiment, it can be assumed that short-term exposure to apigenin in mice causes the development of oxidative stress and triggers an inflammatory cascade characterized by inflammatory cell infiltration and dysfunction of liver cells. As a result of these processes, histopathological changes appear in the liver. Similar observations were made by Singh et al., who after a single intravenous administration of apigenin in mice at a dose of 100 or 200 mg/kg showed an increase in serum AST, ALT, ALP and oxidative stress markers, which led to liver damage. Therefore, the changes in the liver shown in our experiment may indicate a slow process of developing degenerative changes in this organ. Such observations were also made by Alamri (2024), who after 3 weeks of oral administration of apigenin and thioacetamide (TAA) in rats showed mild degeneration and necrosis of liver cells in histological examination. On the other hand, the appearance of such changes after the use of apigenin as subcapsular, single inflammatory focus composed of neutrophils, inflammatory infiltrates composed of mononuclear cells in the subcapsular area, neutrophilic infiltrates in the peripheral zone with reactive stroma small, foci of inflammatory infiltrates composed of lymphocytes in the vicinity of veins suggests that immune cells are recruited to damaged hepatocytes, which is the first step in the process of healing and repairing wounds (Martin and Leibovich, 2005). The role of neutrophils present in the histological image may be phagocytic activity aimed at removing all matrix and cell debris from the damaged liver parenchyma. Therefore, it can be assumed that these negative changes in the liver parenchyma in the first period after the use of apigenin are the result of metabolic and functional changes of this organ in response to the administration of this flavonoid. However, long-term use of apigenin may contribute to the repair of liver tissue, for example, by facilitating the migration of immune cells to the originally damaged regions. This concept is also supported by the lack of changes in the concentration of proinflammatory cytokines in liver tissue and liver enzymes. Therefore, it seems to be worth considering the possibility of pulsatile administration of apigenin to support treatment of liver diseases. However, the validity of this method would have to be verified by further metabolic, molecular, and genetic studies at the cellular level.

Alterations in the liver proteome and structural changes in hepatocytes may suggest potential relevance to human liver conditions such as liver fibrosis, cancer, metabolic and/or inflammatory liver diseases. However, caution should be taken when directly translating these findings to human conditions. In our study, apigenin was given intraperitoneally, whereas oral administration is the most common route for drug delivery in humans. Orally administered apigenin can undergo extensive biotransformation by the intestinal microbiomes. The gut microbiota can metabolize apigenin into other bioactive metabolites that can have different effects. Moreover, apigenin can alter the gut microbiota composition, which in consequence may also influence its metabolism and biological activity (Wang et al., 2019).

This study had some limitations, such as the use of only male mice in order to exclude hormonal effects on the changes observed. Apigenin was used in the experiment for a period of 14 days; however, a long-term experiment should be considered, i.e., at least 3 months of apigenin administration by various routes, including per os, in order to assess its positive or negative effects on the body and the changes it induces in the liver. Chronic exposure studies would be important to determine whether apigenin’s effects on the liver are reversible, progressive, or lead to cumulative organ damage over time. Moreover, our study was carried out on Swiss mice, and while this is a standard animal model, the human applicability of the findings should be strengthened by validating our findigs in human liver cell models or humanized mouse models. Moreover, in order to demonstrate the activation of inflammatory or regenerative processes in liver tissue, the studies should be extended to include the assessment of the activation of inflammatory signaling pathways, such as NF-κB and MAPK, activation of TLR4, or cellular analysis by assessing the activity of Toll-like receptor 4, COX-1, COX-2 enzymes and others that were not determined and that could explain the cellular mechanisms of apigenin’s effect on hepatocytes and its therapeutic usefulness.

## 5 Conclusion

The results demonstrate that exposure of mice to apigenin induces functional and morphological changes in the hepatocytes. These changes lead to an increase in the expression of proteins that participate in limiting inflammatory changes in the liver and in regeneration of the liver. The synthesis of proteins involved in the regulation of cellular metabolism, gluconeogenesis, and maintaining the body’s energy homeostasis was increased as well. The low concentrations of pro-inflammatory cytokines do not confirm the development of systemic inflammatory changes. The absence of changes in absolute and relative liver weights in the group of mice receiving apigenin in comparison to the control group, as well as the unchanged serum concentrations of liver markers such as ALT, AST and ALP, indicates that it has no toxic effect in the body. In order to demonstrate the long-term effects of apigenin on the body, further studies are necessary, including animal models and liver cell lines exposed to apigenin in the course of experimentally induced hepatocellular carcinoma (HCC).

## Data Availability

The original contributions presented in the study are included in the article/Supplementary Material, further inquiries can be directed to the corresponding author.
